# Sexual dimorphism in ritualized agonistic behaviour, fighting ability and contest costs of *Sus scrofa*

**DOI:** 10.1186/s12983-022-00458-9

**Published:** 2022-03-12

**Authors:** Irene Camerlink, Marianne Farish, Gareth Arnott, Simon P. Turner

**Affiliations:** 1 Animal Behaviour and Welfare, Animal and Veterinary Sciences Department, Scotland’s Rural College (SRUC), West Mains Rd, Edinburgh, EH9 3JG UK; 2 Institute of Genetics and Animal Biotechnology, Polish Academy of Sciences, Ul. Postępu 36A, 05-552 Jastrzębiec, Poland; 3 Institute for Global Food Security, School of Biological Sciences, Queen’s University, Belfast, BT9 7BL UK

**Keywords:** Ritualized display, *Sus scrofa*, Behaviour, Aggression, Sex differences, Animal contest, Sexual dimorphism, Fighting ability, Contest costs

## Abstract

**Background:**

Sexual selection has driven sexual dimorphism in agonistic behaviour in many species. Agonistic behaviour is fundamentally altered by domestication and captivity, but it is unclear whether ancestral sex differences remain. We aimed to evaluate the effect of sex on agonistic behaviour, fighting ability and contest costs. We studied this in domestic pigs (*Sus scrofa*) where aggression compromises welfare, and sexual dimorphism in aggression has been inconclusively demonstrated. Behaviour and physiology of 827 male and female juvenile pigs were studied during resident-intruder tests and dyadic contests at various ages, while accounting for the relative body weight difference between the opponents.

**Results:**

Males won in 79% of contests against females, even when at a large weight disadvantage. The effect of sex increased with age, with males having a 138 times higher likelihood of winning than females when 13 weeks old. In dyadic contests, males invested more time in non-damaging agonistic display behaviour and took longer before escalating into damaging aggression. Males showed ritualized display that included foaming from the mouth and piloerection of the neck hair, which was nearly always absent in females. Contest costs in terms of contest duration, blood lactate and skin lesions where higher for males, especially when fighting another male.

**Conclusions:**

Profound sex differences were present for agonistic behaviour, fighting ability and contest costs, and became more pronounced as animals got older. Males invested more in ritualized display before escalating into costly fights, whereas females attacked sooner but also terminated contests more rapidly and with fewer costs. The sexual dimorphism in agonistic behaviour in juvenile domestic pigs is in line with the evolutionary relevance for females’ maternal defence and males’ competition for females.

## Background

Behavioural differences between the sexes are a common phenomenon for most species in the wild, in line with physiological and hormonal differences between males and females [1, 2]. Sex differences may be particularly prominent with respect to agonistic behaviour [3] as sexual selection, typically involving competition between males for breeding opportunities, has increased physical ability (e.g. weaponry) and behaviours that favour success in competition [4, 5].

Across most mammalian species, males are more aggressive than females ([3, 6]; but see Clutton-Brock [7]) and are generally larger [8–10]. Sexual dimorphism can be reflected in fighting ability, or resource holding potential (RHP; Parker [11]), as body weight is often used as proxy measure of RHP (e.g. Briffa and Sneddon [12]). Sex differences also exist in the type of aggression, with males showing more offensive aggression than females [6, 13], and females across species showing more parental aggression related to the defence of offspring (e.g. Elwood et al. [14]). Given the costs associated with aggression, such as energy use, predator attraction and the potential for injury or death, the behavioural repertoire has evolved to minimize costs [15]. In species for which fights can be lethal, elaborate agonistic behaviour, such as threat and submission displays, have evolved [16]. Agonistic behaviour refers to the full repertoire associated with aggression, including ritualized displays, threat and withdrawal, whereas aggression refers to the damaging behaviour within the agonistic repertoire, such as bites and fights [17, 18].

Domesticated animals retain most of the behavioural repertoire of their ancestors, and behavioural changes are rather quantitative than qualitative [19]. Sexual dimorphism in agonistic behaviour of domesticated animals has been studied previously, but there are only a few studies that provide evidence of whether sex differences have been fully conserved (e.g., [20, 21]). In domesticated species, the expression of aggression is influenced by housing and management (e.g. [22, 23]). Group composition may change suddenly and more frequently than in the wild and sexes may be housed together or separated in unnatural ways [24]. Moreover, a small space allowance, and objects and conspecifics that may obstruct movement or sight of the opponent, can hamper proper display of agonistic behaviour. Studies on aggression in captive species largely relate to laboratory mice (e.g. [25]) and farmed pigs (e.g. [26]). Understanding the effect of captive environments on the expression of agonistic behaviour in managed animals is likely to be important to improving their welfare. Although research has led to changes in animal management, aggression is still a serious welfare concern (mice: [27]; pigs: [26]).

Pigs (*Sus scrofa*) and wild boar naturally live in small social groups of adult females and sexually immature males [28] whereas adult males are usually solitary [29]. Females resolve conflicts mainly by threat and withdrawal, and fights are rare. Fights between adult males are costly and injurious and may be lethal, but mostly occur during the breeding season for access to females or when a young unfamiliar male attempts to enter a social group [30]. Domestic pigs show a high motivation to fight and it has previously been suggested that the strong genetic selection on growth performance has increased conspecific aggression as compared to less strongly selected breeds [31], given the genetic association between growth rate and the aggression in pigs [32]. Even hybrid pigs living alongside wild boars exhibit more aggression than their wild counterparts [33]. To date, in pre-pubertal domestic pigs, the literature is ambiguous about whether sex differences in aggressiveness exist [34–36], and males and females both fight intensely to establish dominance relationships when they encounter unfamiliar pigs [37]. The severe group aggression during regrouping [38] is in contrast to the low incidence of aggression within established social groups and the greater sexual dimorphism of adult agonistic behaviour in wild boar. At present it is unclear if the intense aggression in both sexes in domestic pigs is a result of the artificial group mixing context in which it has been studied in captivity [38] and whether the dimorphism remains when studied in a more ecologically relevant context [39]. It is also unknown whether dimorphism increases with age as would be predicted from the marked sex differences in adult wild boar [30].

The aim of this study was to assess sex differences in the expression of agonistic behaviours of pigs at different pre-pubertal ages. We observed 827 male and female pigs during dyadic contests between opponents unfamiliar to each other, thereby providing them with the space to display their full behavioural repertoire. This context is likely to most directly mimic an ecologically relevant scenario. Based on the known sexual dimorphism in adult pigs, we expect pre-pubertal males to differ from females in their fighting ability (RHP) and thus likelihood to win (hypothesis 1). We further hypothesize that males, whose life history has evolved to engage in potentially very costly later life contests for breeding opportunities, show different ritualised agonistic behaviour than females irrespective of age (hypothesis 2). As adult males engage in more costly fights than females, we hypothesize that pre-pubertal males will differ from females in the costs they accrue during dyadic contests (hypothesis 3).

## Results

### Hypothesis 1: males have superior fighting ability

The first hypothesis was that males would have a greater fighting ability (RHP) than females and thus would be more likely to win. From 10 weeks of age onwards males were heavier than females (Table 1, all *p* < 0.001), but not before week 10 (resulting in an interaction between sex and age, *F*_9,3610_ = 4.33, *p* < 0.001). Males did not have a greater body mass index (Table 1).

**Table 1:** Body weight (BW) and Body Mass Index (BMI) in male and female pigs at different weeks of age

	Males	Females	*p* value
BW week 9	29.2 ± 0.31	28.7 ± 0.31	0.27
BW week 10	35.3 ± 0.26	34.0 ± 0.26	0.0007
BW week 11	43.2 ± 0.23	41.5 ± 0.25	< 0.0001
BW week 13	56.5 ± 0.31	54.8 ± 0.31	0.0002
BW week 14	65.2 ± 0.31	62.7 ± 0.31	< 0.0001
BMI week 10	58.3 ± 0.33	58.0 ± 0.36	0.53

Even when relative body weight difference between opponents was accounted for, males had a 16.43 (confidence interval (CI) 8.835, 30.562) times greater odds ratio of winning when they were contesting the opposite sex (χ = 78.1808; *p* < 0.001). Overall, males won 79% of the male–female contests (131/166). At 8, 10, and 13 weeks of age males won 75%, 71% and 87% of the contests, respectively. There was an age by sex interaction (χ = 14.4518; *p* < 0.001), whereby males at 10 weeks of age had a 8.41 times greater likelihood of beating females (CI 3.442, 20.562) but at 13 weeks had a 138.74 times greater likelihood of winning (CI 32.695, 588.741). Although winners were on average larger as compared to the losers (winners + 4.1%, losers − 1.6% of the opponent’s weight, t_265_= − 2.98, *p* < 0.01), relative body weight difference was not a significant predictor of contest outcome when males were contesting females (*p* = 0.28). Nearly one-third (27%) of the males won despite being lighter than the opposing female, even with up to 29% weight disadvantage.

### Hypothesis 2: males make more use of ritualised agonistic behaviour than females

We next hypothesized that males would show more ritualized agonistic behaviour than females, irrespective of age. Across two resident-intruder tests, in which the interaction is terminated as soon as the resident attacks an inferior intruder, females and males attacked the intruder (versus no attack) with a similar frequency (test 1, males (M) 66%, females (F) 70% attacked the intruder; *p* = 0.28; test 2, M 72%, F 77% attacked; *p* = 0.33). Attack latency did not differ between males and females that did attack in the first test (*p* = 0.58) while females tended to attack the intruder faster in their second test compared to males, which was just outside the threshold for statistical significance (M 77 ± 3.2 s, F 68 ± 3.3 s; *F*_1,547_ = 3.85, *p* = 0.050). There was no effect of age and no sex × age interaction on whether residents attacked, or their attack latency (all *p* > 0.05).

In dyadic contests, 47 males and 37 females had an inconclusive outcome in which no winner could be established within the maximum time, and 57% of the inconclusive contests were at 13 weeks of age. From the male–female contests, females more often initiated an attack (bite initiation: M 57 times, F 78 times; *F*_1,146_ = 5.53, *p* = 0.02). Attacks were less often initiated at 13 weeks of age (33 times) as compared to 8 weeks of age (74 times) (*F*_2,146_ = 6.00, *p* = 0.003), (but there was no age by sex interaction (*p* = 0.23).

Considering all contests, males were involved more often in contests that escalated to a fight (70% of males and 54% of females; beta estimates (*b*): M 0.83, F 0.23; *F*_1,9_ = 10.30, *p* = 0.01). However, this was fully explained by inter-male aggression, with 81% of the male–male contests (MM) escalating into a fight, whereas only 57% of male–female contests (MF) and 52% of female–female contests (FF) escalated (*b*: MM 1.37, MF 0.31, FF 0.14; *F*_2,7_ = 9.58, *p* = 0.01). Contest escalation strongly depended on age, with only 37% of contests escalating when pigs were 13 weeks of age (W), whereas at 8 and 10 weeks this was 77% and 74%, respectively (*b*: W8 1.21, W10 1.00, W14 -0.39; *F*_2,709_ = 25.98, *p* < 0.001). There was no sex × age interaction for fight escalation (*p* = 0.44).

**Fig. 1:**
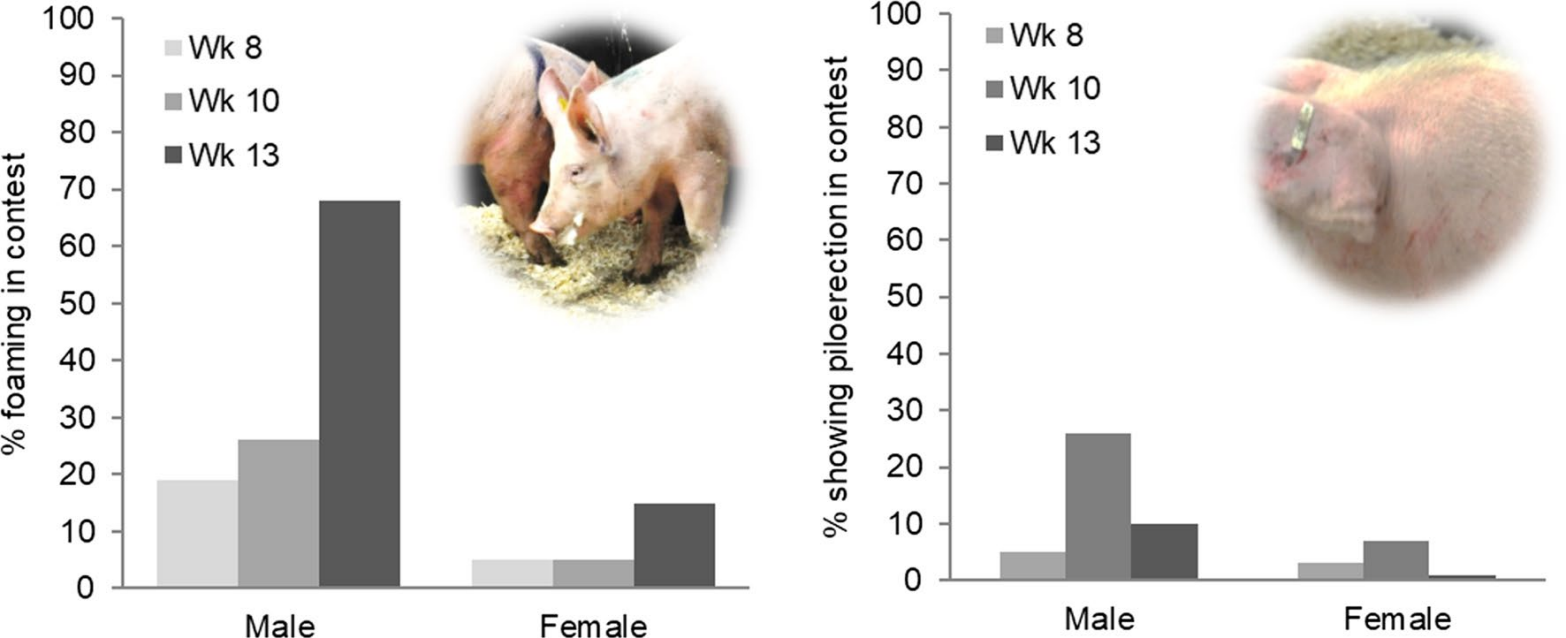
Foaming of the mouth and piloerection of neck hairs in males and females during dyadic contests at 8, 10 and 13 weeks of age (n = 600 animals). Photos by M. Farish

Males took longer before engaging in the damaging contest phase of biting and fighting (Table 2). Older opponents also took longer before they engaged in agonistic display and pushing, and before they withdrew (Table 2). Foaming from the mouth and piloerection of the neck hairs occurred almost exclusively in males (sex effect foaming *b*: M − 0.35, F − 2.43, *F*_1,9_ = 61.19, *p* < 0.001; piloerection *b*: M − 2.52, F − 4.25, *F*_1,9_ = 9.86, *p* = 0.02) and foaming increased with age (foaming: *F*_1,9_ = 35.23, *p* < 0.001; piloerection: *p* = 0.35) (Fig. 1).

**Table 2:** Contest behaviour for contests that ended in a clear winner within 20 min

Latency (s)	8 weeks		10 weeks		13 weeks		*p* value		
	Male	Female	Male	Female	Male	Female	Sex	Age	S × A
Nose contact	10.8	10.3	17.4	19.7	12.5	10.9	0.35	< 0.001	0.07
Display	30.3	36.5	37.4	54.1	37.4	57.0	0.38	0.03	0.92
Pushing	57.9	70.9	64.7	125.9	91.7	103.7	0.57	0.02	0.81
Bite	112.9	99.1	149.7	113.4	157.0	92.6	0.01	0.57	0.41
Fight	108.9	90.5	153.7	78.2	159.3	86.4	0.002	0.14	0.14
Withdrawal	161.4	156.8	191.8	168.2	349.5	127.5	0.93	0.008	0.07

Values are the mean latency (in s) until a behaviour was shown within dyadic contests, by week of age for male and female pigs. Values are means to facilitate interpretation, whereas the *p*-values are based on log transformed data

**Fig. 2:**
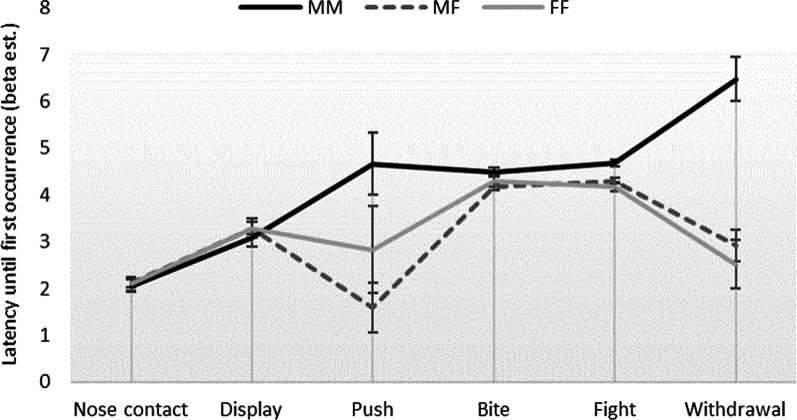
Mean latency until the first occurrence of the observed behaviours for dyadic contests between males (MM, black solid line), males and females (MF, dark grey dotted line) and females (FF, light grey solid line). Values are the beta estimates (LSmeans) of the log transformed latencies with their standard errors

The sex of the opponent influenced the behaviour, with male–male contests (MM) having the longest latency before withdraw as compared to MF and FF contests (*b*: MM 6.48, MF 2.92, FF 2.51; *F*_2,3_ = 16.51, *p* = 0.02), but there was no difference in the latency of the other behaviours (Fig. 2). Inter-male contests had the greatest occurrence of foaming (MM 45.7%, MF 17.4%, FF 7.2%; *b*: MM − 0.01, MF − 1.81, FF − 2.29; *F*_2,7_ = 15.12, *p* = 0.003), but not piloerection (*p* = 0.20).

### Hypothesis 3: males accrue greater contest costs

We further hypothesized that males would accrue more costs than females during contests. Males indeed had a longer contest duration, a greater proportional increase in blood lactate during the contest, and more skin lesions (as a result of being bitten) on the front of the body (Table 3). Contest duration, blood lactate and blood glucose most strongly increased in contests at 10 weeks of age. For contest duration and skin lesions on the front of the body the effect of sex interacted with age, with least costs accrued by females at 13 weeks of age (Table 3). When taking into account the sex of the opponents, male–male contests showed the greatest accumulation of costs (Table 4).

**Table 3:** Measures of contest costs for males and females at 8, 10 and 13 weeks of age

	8 weeks		10 weeks		13 weeks		*p* value		
	M	F	M	F	M	F	Sex	Age	S×A
Contest duration (s)	267	242	375	335	311	184	0.004	0.003	0.04
Δ Lactate (mmol/L)	4.65	4.00	5.60	4.91	4.65	2.70	0.009	0.02	0.18
Δ Glucose (mmol/L)	1.20	1.14	1.21	1.20	1.14	1.09	0.12	0.03	0.64
Skin lesions front (n)	24	26	25	18	30	17	0.02	0.58	0.03
Skin lesions total (n)	37	43	43	33	47	32	0.17	0.92	0.05

Values are LSmeans. For blood lactate and blood glucose, the relative difference between the pre and post contest value was used for analysis

**Table 4:** Measures of contest costs for contests between males (MM), between males and females (MF) and between females (FF)

	MM	MF	FF	*p*-value
Contest duration (s)	349 ± 18.3^a^	264 ± 11.9^b^	258 ± 19.1^ab^	0.01
Δ Lactate (mmol/L)	5.57 ± 0.359^a^	3.82 ± 0.234^b^	4.29 ± 0.375^ab^	0.01
Δ Glucose (mmol/L)	1.21 ± 0.023^a^	1.15 ± 0.015^a^	1.15 ± 0.024^a^	0.16
Skin lesions front (n)	35 ± 2.5^a^	18 ± 1.6^b^	21 ± 2.6^b^	0.002
Skin lesions total (n)	58 ± 4.2^a^	31 ± 2.7^b^	33 ± 4.4^b^	0.002

^a,b^Values lacking a common superscript letter differ by *p* < 0.05 in post-hoc test with Bonferroni correction for multiple testing

## Discussion

This study confirmed profound sex differences in agonistic behaviour in juvenile pigs. Specifically, it showed that male pigs have a greater fighting ability (i.e., RHP), differ in their expression of agonistic behaviour and accrue more costs from aggression as compared to females. Females more often initiated the attack in the dyadic contest, but were considerably less likely to escalate into fights. At 13 weeks of age, females had the lowest costs from the interaction with an opponent. The sexual dimorphism increased as pigs got older.

### Fighting ability

Body weight and size are often used as a proxy measure for RHP [12]. Weight differences between pre-pubertal male and female pigs are minimal, whereas at adult age female sows (of a commercial white type breed) weigh 250–350 kg while adult boars of the same type weigh 300–450 kg. According to our results, the sexual size dimorphism becomes significant from 10 weeks of age, while the body mass index at this age did not differ between the sexes. Surprisingly, sex fully overruled any body weight advantages in the likelihood of winning when males fought females. Previous studies showed that heavier pigs are more likely to win [34, 37, 40], which holds true for same-sex contests. However, in male–female contests, regardless of the weight difference between the opponents, males nearly always outcompeted females, especially at 13 weeks of age. The clearly greater RHP of males was regardless of body weight. Therefore, RHP likely includes components other than body weight, for example personality [41, 42] or motivation to persist in the encounter [43, 44]. Previous experience also contributes to the likelihood of winning, as reflected in winner-loser effects (Hsu and Wolf [45]; pigs: Oldham et al. [46]). As young male piglets are known to engage more than females in play fighting behaviour [47, 48], which can substitute for real fighting [49], this may already provide them with some experience of victory as well as training for future encounters. Especially for encounters without physical contact, traits related to persistence capacity may be valuable alternatives or additions to the traits related to physical strength [50]. Overall, a multivariate measure of RHP may better capture the individual’s fighting ability [50, 51].

### Agonistic behaviour

Males may have evolved to use agonistic behaviour in a different context than females. As adults, male wild boar are solitary and fight fiercely with unfamiliar males to obtain access to females [29, 30]. Males in our study waited longer before engaging in damaging behaviour and showed more foaming from the mouth and piloerection of the neck hair, which has also been described for wild boar males during competition for females [29]. The longer and more elaborate display behaviour may be a strategy to avoid costs. This potential cost-reducing benefit of display behaviour was not present in unexperienced males, for whom most contests despite display escalated into a fight. Only at 13 weeks of age, after contest experience was gained, did contest escalation reduce. Pigs indeed require a substantial fighting experience—not merely experience of early life play fighting [52]—before they become proficient in mutual assessment [37]. Therefore the benefit of longer ritualized pre-escalation display may only appear after obtaining repeated fighting experience. Overall, males seem to take longer to decide whether to engage in damaging aggression. The sex differences were especially apparent in inter-male contests, which were more intense and costly. The dyadic male–male contests most closely relate to the natural situation between adult individuals, and the aggression shown in this scenario may be a relevant reflection of natural agonistic behaviour in domestic pigs.

Females in nature live in small matriarchal family groups in which subordinates avoid aggressive conflict (wild boars and feral pigs; [33, 53, 54]) and their aggression relates mainly to offspring defence. Their responses need to be rapid in order to defend the piglets. The rapid bite initiation in the dyadic contests is in line with other studies [55, 56], although others have found no effect of sex when comparing females with entire (not castrated) males [57]. It has been suggested that aggression in female juvenile pigs may have increased with the commercial push for larger litter size, as the smaller female piglets may face stronger early-life postnatal competition for nutrition [58]. Females from litters with a higher male:female sex ratio may also develop more aggressive behaviour [59].

### Contest costs

The changes in blood metabolites and skin lesions showed that males invested more heavily in the contests, especially when facing a male opponent. Males had a greater increase in blood lactate than females, but not a significantly greater increase in blood glucose than females. Lactate relates to contest intensity in both winners and losers, whereas the increase in glucose may be more profound in losers [60]. Males had more skin lesions on the front of the body than females, but not in total. This provides evidence of males proactively engaging in frontal attacks. Skin lesions on the middle and especially rear section of the body are typically related to the receipt of unilateral aggression such as bullying [61]. Contest costs were highest at 10 weeks of age. The lower contest costs at 13 weeks of age may be related to the experience that the animals have gained from earlier contests, as contest experiences may alter their opponent assessment strategies, resulting in opponents terminating the contest without engaging in costly fights [37, 62].

### Context in which agonistic behaviour occurs

Previous findings regarding pre-pubertal male and female pig aggression that contrast with those reported here may be due to the commonly used artificial test situations that are largely unnatural (e.g., encounters between groups of unfamiliar animals; [38]) or do not provide the opportunity to display the full behavioural repertoire or to withdraw (e.g., due to restricted housing conditions; [63]). The current experimental design may better reflect the ecologically relevant scenario in which opponents would encounter each other in nature.

While existence of sexual dimorphism in agonistic behaviour of wild and feral populations of pigs is known for adult animals [28, 64], including differences in body size and social behaviour, juveniles of such populations have not been studied for sexual dimorphism in agonistic behaviour. It can therefore not be concluded whether the dimorphism reported here has resulted from natural selection or more recent domestication and selective breeding. However, the ritualized display seen in the domestic juveniles is similar as described for adult wild boar males [29]. Due to genetic selection domestic females attain puberty at an average of 6.5 months [65] instead of at ca. 8–10 months as seen in female wild boars [64, 66]. The earlier onset of puberty may have encouraged sexual dimorphism in aggression at a younger age in domestic pigs than would be case in nature. The results of the current study relate to pre-pubertal pigs and present novel evidence of clear sex differences in early life aggression. Such differences may relate to the differing life history strategies of the sexes, with males on a developmental trajectory to engage in future escalated contests for access to breeding females, whereas females engage in less costly aggressive encounters in later life [64].

## Conclusions

Under a test condition that provided animals with the space and relevant context to express their behaviour, males differed profoundly from females in their fighting ability, agonistic behaviour and the associated costs, despite their prepubertal age. Males invested more in ritualized display before escalating into costly fights, whereas females attacked sooner but also terminated contests more rapidly and with fewer costs. The sexual dimorphism in agonistic behaviour in juvenile domestic pigs is in line with the evolutionary relevance for females’ maternal defence and males’ competition for females.

## Methods

This work uses the combined data of three separate but similar studies, where the herd, management, tests and procedures were the same. Details of the studies, other than described below, can be found in Camerlink et al. [37, 42, 67]. The protocols were approved by SRUC’s animal experiments committee and were carried out under UK Home Office license (project licence PPL60/4330), and in constant collaboration with SRUC’s named veterinary surgeon.

### Experiments

Across three experiments, 438 males and 389 females were studied (total n = 827). Experiment 1 aimed to assess the influence of aggressiveness as a personality trait on later dyadic contest behaviour (for details see [42]). The trial included 136 individuals (Table 5), studied over three separate batches (i.e. farrowing groups) in 2014. Experiment 2 focussed on the influence of aggressiveness as a personality trait and experience of regrouping on contest behaviour. Male and female pigs (n = 311) were studied over four batches from Nov 2014–Nov 2015. The regrouping experience, described in detail in Camerlink et al. [37] influenced contest decision making and has been accounted for in the statistical models of the current study by including experiment number as random variable. Experiment 3 determined the effect of early life socialization of piglets (n = 380) on contest behaviour over six batches in 2016. Socialization aims to provide pigs with better social skills [67]. From two weeks of age, 50% of the sows with piglets were socialized up to weaning, meaning that the piglets of two neighbouring litters could mingle freely between the two pens. Socialization reduced attack latency and contest duration [67] and was accounted for in the statistical models by including experiment number as random variable. An overview of the differences between the experimental designs is provided in Table 5.

**Table 5:** Overview of the experimental design and timeline of the three experiments (exp.)

Design	Exp 1	Exp 2	Exp 3
N males	68	153	217
N females	68	158	163
Socialization (week of age)	n/a	n/a	2
Resident-Intruder test (week of age)	9	9	7
Dyadic contest 1 (week of age)	10	10*	8
Treatment contest 1	RHP match	RHP match /not	RHP match/not; socialized/not
Regrouping (week of age)	n/a	12	n/a
Dyadic contest 2 (week of age)	n/a	13	n/a
Treatment contest 2		RHP match/not; regrouped/not	

*Excluded from analyses to avoid repeated observations for animals

### Animals and housing

Male and female pigs (originating from Large White × Landrace sows sired by American Hampshire boars) were studied at the SRUC research farm (Easter Howgate, UK). Males were not castrated and the tail and teeth of all pigs were kept intact. Cross-fostering was applied if the number of piglets exceeded the number of functional teats, and this may for the fostered piglets result in early experience in competition for nutrition [68]. Piglets had been raised in farrowing crates and were weaned at four weeks of age. After weaning they were kept with their litter groups in pens measuring 1.9 × 5.8 m (~ 1.0–1.1 m^2^/pig). Pens had a solid floor which was covered with approximately 5 kg of long straw, and were cleaned daily and provided with ~ 3.5 kg of fresh straw. There was *ad libitum* access to water and pelleted feed, and they were never feed restricted. From 6 weeks of age they were gradually habituated to the test situation to reduce the possibility of fear responses during any of the tests. For the habituation, each group of pigs was exposed three times to each test situation (weigh scale, walking in the corridor, and moving to the test arena), whereby the group size was reduced from 50% to ca. 30% to moving pigs alone. If they showed three times signs of distress (loud vocalizations, escape attempts), they were returned to the home pen. Pigs that failed to complete two of the three sessions were excluded from the tests. Body weight was measured at weaning and in the week before contests. In experiment 1 and 2, body conformation was assessed at ten weeks of age by measuring the circumference of the rib cage and the body length from crown to tail base. For 447 pigs, a body mass index (BMI) was calculated as [body weight/crown-rump length^2^] [69].

### Resident-intruder tests

Animals were subjected to the resident-intruder (RI) test to obtain a measure of individual aggressiveness (details provided in Camerlink et al. [42]). Each focal animal (a ‘resident’) was temporarily held in a separate part of its home area, at which stage an unfamiliar inferior (lighter weight) ‘intruder’ of either sex entered the resident’s area. The latency until attack (of either resident or intruder) was recorded, as well as the number of times either the resident or intruder mounted the other (standing with both front legs on the body of the other). The test was terminated when one of the animals bit the other, which provides the main variable of the attack latency [55]. To avoid unnecessary harm to the animals, the test also ended when the resident did not attack within 5 min after initial contact; when an animal was mounted five times; or when one of them showed a clear fear response (repeated vocalizations for 1 min, or after three attempts to escape the test area). The test was repeated the following day with a different intruder per resident. The intruders only participated in the RI test and were not part of the further trials. Residents were never used as intruders. The variables used from the RI test were ‘attack’ (yes/no) and the attack latency (in seconds) for test day 1 and 2 (day 1 and 2 analysed separately).

### Dyadic contests

In dyadic contests, our proxy measure of RHP was body weight, with opponents either matched for body weight (< 5% difference) or not (> 15% difference). Opponents were unfamiliar to each other. Males and females were paired to create male–female (MF), male–male (MM), and female–female (FF) dyads. Contests took place in a novel and neutral test arena (2.9 × 3.8 m) without resources (e.g. no feed). Opponents received a mark on their back (using Raidex animal marker spray) for individual recognition. Behaviour was recorded live (described below). Contests were ended when a clear winner was apparent, indicated by a head-tilt movement of the loser and no retaliation within 1-min after being attacked. To reduce the aversive impact of fights on animal welfare, contests were terminated if after 20 min no clear winner was present, or in the case of a repeated fear response (three escape attempts or 1 min continuous loud vocalizations), or three occurrences of mounting. These end-points were the same across the experiments, and prevented contests in which one or both of the opponents were unwilling to engage in agonistic behaviour. Behaviour was live observed by a single observer using the ethogram in Table 6. For these behaviours, with the exception of foaming and piloerection, the initiator at the first occurrence of the behaviour was noted. ‘Contest duration’ refers to the duration that the opponents spent in the contest arena, whereas ‘fight duration’ refers to engagement in mutual damaging aggression as described in Table 6. The observer noted the latency until the first occurrence of each agonistic behaviour, and whether foaming of the mouth or piloerection of the neck hair occurred at any time during the contest.

**Table 6:** Ethogram of behaviours recorded during the dyadic contests

Behaviour	Measure	Description
Nose contact	Latency	Nose approaches within 5 cm of the snout of the opponent
Display	Latency	Parallel walking (move simultaneously with the shoulders next to each other); heads up (both have their nose lifted high up in the air alongside each other); or shoulder-to-shoulder (standing or moving with the shoulder against the shoulder of the opponent without real pressure)
Mutual pushing	Latency	Head or shoulder is used to move the opponent aside by applying pressure
Unilateral bite	Latency	Opens mouth and delivers a bite that contacts the opponent
Fight	Latency	Rapid sequence of bites which are retaliated with a similar aggressive act from the opponent within 5 s
Withdrawal	Latency	Turns its head away from the opponent and retreats from further attacks by not showing any aggressive behaviour within 10 s
Foaming	Yes/no	Froth appears from the mouth due to repeated teeth grinding
Piloerection	Yes/no	Hairs in the neck are raised as compared to their normal flat position

### Measures of contest costs

As contest duration does not necessarily reflect contest intensity, several measures of costs were taken. Just before and after each contest, pigs were sampled for blood glucose and blood lactate by using a flat blade lancet to produce a drop of blood from an ear vein. The drop of blood was immediately applied onto a test strip of a glucose meter (IME-DC iDia) and a test strip of a lactate meter (The EDGE Lactate Analyser). For details see Camerlink et al. [42]. The number of skin lesions resulting from bites is a validated proxy measure for the amount of aggression received [61]. Skin lesions were counted for each individual on the front (head and shoulders), middle, and rear (from hind legs to tail) before and directly after each contest. Contest costs were analysed as contest duration (minutes), relative change in blood glucose (mmol/L), relative change in blood lactate (mmol/L), the number of skin lesions on the front of the body, and the total number of skin lesions on the body. The number of skin lesions present pre-contest was subtracted from the number counted post-contest.

### Statistical analyses

Data were analysed with SAS version 9.4 (SAS Institute Inc, Cary, USA). Data of the three experiments were collected in the same manner and were merged into one data set. The final data set contained 201 female–female (FF) dyadic contests, 246 male–male (MM) contests and 373 male–female (MF) contests. While the hypotheses are stated in the direction of males showing greater fighting prowess than females, the opposite can potentially occur, and therefore two-sided tests were used.

#### Analysis for hypothesis 1: males have superior fighting ability

Body weight (kg) was analysed in a mixed model with the categorical variables sex (M/F), age (8, 10 or 13 weeks) and their interaction as predictor variables. Pig ID was included as repeated effect to account for multiple observations over time on the same animals. The sex difference in BMI was analysed by a t-test with BMI at 10 weeks of age as a normally distributed continuous value and sex as class effect. Likelihood of winning was analysed in a logistic model for male–female (MF) contests, with contest outcome as response variable and sex, age, relative body weight difference as compared to the opponent and the interaction between sex and age as predictor variables.

#### Analysis for hypothesis 2: males make more use of ritualised agonistic behaviour than females

Attack (yes/no) for test 1 and test 2 in the RI-test was analysed in a binary model (proc GLIMMIX with binary distribution and logit link function) with sex, age and their interaction as predictor variables and treatment (Control/Regrouped/Socialized) nested within experiment (Exp 1, 2, or 3) as a random variable to account for differences between the three experiments. RI test attack latency at test 1 and 2 was analysed in mixed models with sex, age and their interaction as predictor variables and treatment nested within experiment as a random variable.

For dyadic contest behaviour, inconclusive contests (i.e., contests in which no winner emerged within the 20 min time limit, regardless of interaction) were removed (42 contests omitted: 6, 8, and 7% of contests in Exp. 1, 2 and 3, respectively), which included contests lasting 1200 s (20 min; 9 contests omitted). Latencies until each level of escalation were log transformed due to a non-normal distribution of the residuals, and analysed as response variables in separate mixed models, with sex, age and their interaction, and whether opponents were weight matched (yes/no) as predictor variables. Treatment (Control/Regrouped/Socialized) nested within experiment (Exp. 1, 2, or 3) was included as a random variable. The contest dyad was specified as subject (n = 166) to account for dependence between the opponents within the contest. In an alternative model, the sex combination of the opponents (FF, FM, MM) was added in addition to the original model to assess the influence of the opponent’s sex. The binary variables bite initiation (only for male–female contests), fight occurrence, foaming and piloerection (for exp 2 and 3) were analysed as in the original and alternative model for the latencies, but using the GLIMMIX procedure with a binary distribution and logit link.

#### Analysis for hypothesis 3: males pay greater contest costs

Variables related to contest costs (blood glucose, blood lactate, contest duration, and skin lesions) were analysed in mixed models (Proc MIXED) as response variables. Sex, age and their interaction, and weight matching were the predictor variables. The sex combination of the opponents (FF, FM, MM) was added in an alternative model in addition to the other variables. Treatment nested within experiment was included as a random effect, and the dyad was specified as the subject.

For the mixed models for hypothesis 2 and 3, post hoc tests were adjusted with the Bonferroni correction to adjust for multiple testing. The residuals of all models were assessed for their approximation to the normal distribution. Data are presented as means and standard error (SE), unless stated otherwise. *P*-values < 0.05 were considered significant.

## Data Availability

The datasets used and/or analysed during the current study are available from the corresponding author on reasonable request.
